# Recent Advances in Molecularly Imprinted Polymers for Antibiotic Analysis

**DOI:** 10.3390/molecules28010335

**Published:** 2023-01-01

**Authors:** Guangli Zhao, Yue Zhang, Dani Sun, Shili Yan, Yuhao Wen, Yixiao Wang, Guisheng Li, Huitao Liu, Jinhua Li, Zhihua Song

**Affiliations:** 1 School of Pharmacy, Collaborative Innovation Center of Advanced Drug Delivery System and Biotech Drugs in Universities of Shandong, Key Laboratory of Molecular Pharmacology and Drug Evaluation (Yantai University), Ministry of Education, Yantai University, Yantai 264005, China; 2 CAS Key Laboratory of Coastal Environmental Processes and Ecological Remediation, Shandong Key Laboratory of Coastal Environmental Processes, Shandong Research Center for Coastal Environmental Engineering and Technology, Yantai Institute of Coastal Zone Research, Chinese Academy of Sciences, Yantai 264003, China; 3 College of Chemistry and Chemical Engineering, Yantai University, Yantai 264005, China; 4 Shandong Zafex Scientific Instrument Co., Ltd., Rushan 264599, China

**Keywords:** antibiotics, molecularly imprinted polymers, imprinting techniques, solid-phase extraction, sensor

## Abstract

The abuse and residues of antibiotics have a great impact on the environment and organisms, and their determination has become very important. Due to their low contents, varieties and complex matrices, effective recognition, separation and enrichment are usually required prior to determination. Molecularly imprinted polymers (MIPs), a kind of highly selective polymer prepared via molecular imprinting technology (MIT), are used widely in the analytical detection of antibiotics, as adsorbents of solid-phase extraction (SPE) and as recognition elements of sensors. Herein, recent advances in MIPs for antibiotic residue analysis are reviewed. Firstly, several new preparation techniques of MIPs for detecting antibiotics are briefly introduced, including surface imprinting, nanoimprinting, living/controlled radical polymerization, and multi-template imprinting, multi-functional monomer imprinting and dummy template imprinting. Secondly, several SPE modes based on MIPs are summarized, namely packed SPE, magnetic SPE, dispersive SPE, matrix solid-phase dispersive extraction, solid-phase microextraction, stir-bar sorptive extraction and pipette-tip SPE. Thirdly, the basic principles of MIP-based sensors and three sensing modes, including electrochemical sensing, optical sensing and mass sensing, are also outlined. Fourthly, the research progress on molecularly imprinted SPEs (MISPEs) and MIP-based electrochemical/optical/mass sensors for the detection of various antibiotic residues in environmental and food samples since 2018 are comprehensively reviewed, including sulfonamides, quinolones, β-lactams and so on. Finally, the preparation and application prospects of MIPs for detecting antibiotics are outlined.

## 1. Introduction

Antibiotics are a class of secondary metabolites produced by bacteria, mycetes or other microorganisms over the course of their lives that inhibit pathogenic or other biological activity and are widely used not only for the prevention and treatment of human and animal diseases but also as growth promoters in animal husbandry and aquaculture [1]. The mainly used antibiotics include sulfonamides (SAs), quinolones (QNs), β-lactams (BALs), macrolides (MALs), tetracyclines (TCs), aminoglycosides (AGs) and others [2]. The discovery and use of antibiotics has brought hope to humans and animals in the fight against many infectious diseases [3], but the increased misuse of antibiotics has led to residues in animal products, groundwater and soil, which has had a considerable impact on human health; for example, they can lead to bacterial resistance and allergic reactions, which has caused concern worldwide [4]. Therefore, there is an urgent need to establish effective methods for the analysis of antibiotic residues. However, the low residue levels of antibiotics, the many interfering factors and the complexity of the samples make the selective enrichment of targets before detection crucial [5].

The purpose of sample pretreatment is to separate and preconcentrate the target analytes and eliminate the matrix effects [6]. Sample pretreatment has become a decisive step in the analytical determination of trace targets in complicated matrices, which not only improves analytical sensitivity and accuracy but also reduces instrument maintenance and operating costs [7]. Sample pretreatment techniques include liquid phase microextraction, supercritical fluid extraction and solid phase extraction (SPE), etc. [1]. Among them, SPE is a common sample pretreatment technique. This is because it has the advantages of being less time coming, simple to operate and efficient, with a low cost, low consumption of organic solvents and high enrichment efficiency [8]. SPE mainly includes packed SPE (PSPE), magnetic SPE (MSPE), dispersive SPE (DSPE), matrix solid-phase dispersion (MSPD), solid-phase microextraction (SPME), stir-bar sorptive extraction (SBSE) and pipette-tip SPE (PT-SPE). Conventional SPE has problems, such as low filler selectivity, long extraction times and complex operation. Therefore, it is crucial to find new fillers [9].

For the detection of antibiotics, there are methods, such as chromatography [10], mass spectrometry (MS) [11] and sensor detection [12]. Chromatography mainly includes high-performance liquid chromatography (HPLC) and liquid chromatography coupled with tandem MS (HPLC-MS/MS). Sensors include electrochemical, optical and mass-sensitive sensors. Sensor detection technology has become a hot spot in detection technology research because of its high sensitivity, fast detection speed and other advantages. Specific sensing materials have an important impact on the sensitivity and selectivity of the sensor [13]. However, the antibody of the sensor is usually unstable; there is an urgent need for a stable antibody as a recognition element of the sensor for the detection and analysis of antibiotics in complex substances [13,14].

**Figure 1:**
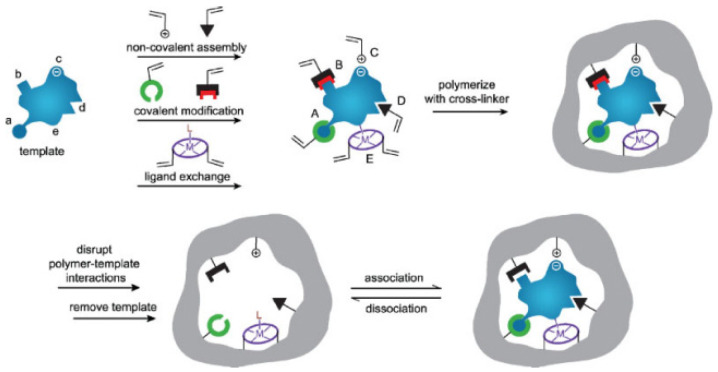
Highly schematic representation of the molecular imprinting process. The formation of reversible interactions between the template and polymerizable functionality may involve one or more of the following interactions: [(**A**) reversible covalent bond(s), (**B**) covalently attached polymerizable binding groups that are activated for non-covalent interactions via template cleavage, (**C**) electrostatic interactions, (**D**) hydrophobic or van der Waals interactions or (**E**) co-ordination with a metal center, each formed with complementary functional groups or structural elements of the template, (**a**–**e**) respectively]. Subsequent polymerization in the presence of crosslinker(s), a cross-linking reaction or other process results in the formation of an insoluble matrix (which itself can contribute to recognition through steric, van der Waals and even electrostatic interactions) in which the template sites reside. The template is then removed from the polymer through the disruption of polymer—template interactions and extraction from the matrix. The template, or analogues thereof, may then be selectively bound again by the polymer in the sites vacated by the template, the ‘imprints’. While the representation here is specific to vinyl polymerization, the same basic scheme can equally be applied to sol-gel, polycondensation, etc. [16].

MIPs are prepared through molecular imprinting technology (MIT), which is often depicted as a technique for creating an “artificial lock” that identifies a “molecular key” by simulating enzyme–substrate or antigen–antibody interactions [15]. As schematically represented in Figure 1 [16], the preparation process of MIPs is usually divided into three parts: preassembly or copolymerization of the template molecule with the functional monomer via certain means (e.g., non-covalent hydrogen bonding, electrostatic forces, hydrophobic forces, covalent bonding, coordination, etc.) to form the host–guest complex; addition of a crosslinker and initiation through certain initiation methods (e.g., initiator, thermal initiation, photoinitiation, etc.) to produce a highly cross-linked polymer; the template molecules are removed through appropriate means to obtain MIPs with the conformationally predetermined and specific recognition of the template molecules [17,18,19,20]. MIPs have been widely used in sample pre-treatment, chromatographic separations, sensors, etc. MIPs are of interest for their high selectivity, chemical stability, ease of preparation and low cost [21]. Their use as fillers for SPEs and recognition elements for sensors is the most commonly used technique for sample pre-treatment [22,23] and sensor detection [18]. They have been widely used for antibiotic residue analysis.

Therefore, we purpose to review recent advances in MIPs in antibiotic analysis since 2018, focusing on molecularly imprinted SPE (MISPE) and MIP-based sensor applications. The commonly used emerging techniques for the preparation of MIPs for detecting antibiotics, the classification of SPE and the principles of sensors are introduced. Then, the application of MISPE in combination with chromatographic determination and MIP-based sensors for antibiotic detection are outlined. Finally, the perspectives of MIP preparation and SPE/sensing applications are proposed.

## 2. Emerging Techniques for the Preparation of MIPs

In general, various modes of MIT have been increasingly developed and applied, including surface imprinting, nanoimprinting, living/controlled radical polymerization (LCRP) technology, multi-template, multi-functional monomer and dummy template imprinting strategies [23,24,25], and using these, a variety of high performance MIPs targeting antibiotics have been prepared.

Surface imprinting technology means that MIPs are usually prepared as a layer on hard particles, forming recognition sites with high affinity on the substrate surface [26,27]. The uniformly distributed sites not only increase the adsorption capacity of the MIPs and improve the rates of rebinding of the recognition sites to the imprinted molecules but also enhance the adsorption and separation efficiency of the imprinted material [28,29]. All binding sites are confined to the surface, facilitating the elution and reconstitution of template molecules. In addition, this technology allows effective control of the size of the imprinting cavity on the polymer surface [30].

Nanoimprinting technology is used for the preparation of nanostructure MIPs and offers the advantages of high resolution, fast processing speed, high throughput, compatibility with a wide range of materials and low cost, making the technique suitable for the large-scale production of antimicrobial surfaces based on a variety of polymer surfaces [31]. Nanoimprinting materials are expected to improve the adsorption capacity, rebinding kinetics and site-accessibility of MIPs [32].

LCRP technology is increasingly used for MIP preparation for the detection of antibiotic residues and other environmental pollutants [33], of which nitroxide-mediated free radical polymerization and atom transfer radical polymerization (ATRP) and reversible addition-fragmentation chain transfer (RAFT) polymerization are the three most commonly used polymerization methods. Their advantages are mainly reflected by: (1) a wide range of polymerizable monomers, controllable polymer molecular weight and narrow molecular weight distribution; (2) mild reaction conditions, the polymerization reaction temperature is low and can be carried out in a variety of solvents; (3) functional control of the structure, the use of “reactive” features and functionalized end groups allows for the preparation of polymers with complex compositions and structures; (4) the molecular weight of the polymer increases linearly with the conversion rate [34].

A multi-template imprinting strategy means using multiple target molecules as templates, and thereby, multiple types of recognition sites are generated in a single polymeric material. The MIPs allow for the simultaneous extraction, isolation, analysis and detection of different classes of species, greatly broadening the practical applications of MIPs. The simultaneous recognition of multiple target molecules by multi-template MIPs is highly advantageous for the concept of sustainable development [18,25].

A multi-functional monomer imprinting strategy, which involves the use of non-covalent bonds between two or more functional monomers and the template molecule to form different forces with selective adsorption ability, improves the selectivity of MIPs for the template molecule and thus raises the enrichment capacity [35].

A dummy template imprinting strategy, also known as a virtual template imprinting strategy, has been increasingly used in recent years by using structural analogues of the target compound as template molecules, which can be substituted when the target compound is not suitable for use as a template molecule or when the target compound is prone to degradation [36,37].

## 3. SPE Modes Based on MIPs

**Figure 2:**
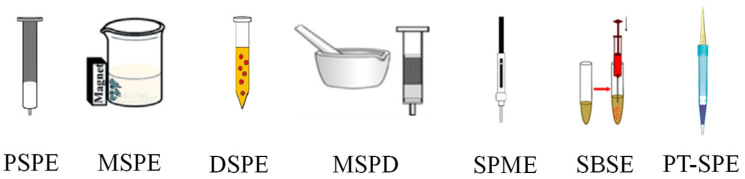
Schematic illustration of the different modes of SPE. Adapted from Ref. [39].

SPE is based on the principle of transferring the target from the aqueous phase to the active center of the adjacent solid phase and has become a commonly used enrichment technique. When low concentrations of analytes are recovered, the use of highly selective MIPs as solid sorbents for SPE not only enables efficient extraction of the target analyte but also increases the enrichment capacity [38], and therefore, the technique has also been widely used for residue detection of antibiotics [39]. A schematic illustration of several different modes of SPE based on MIPs is shown in Figure 2, adapted from Ref. [39], and are briefly described below, namely PSPE, MSPE, DSPE, MSPD, SPME, SBSE and PT-SPE.

PSPE is a common method for sample pretreatment, mainly using solid sorbents to adsorb target compounds in liquid samples, which are then eluted by chemical reagents or resolved through heating to achieve separation and enrichment of the target compounds. The use of MIPs as a solid sorbent can greatly improve the extraction efficiency of PSPE due to its specific recognition ability and high resolution [40,41]. Chen et al. [42] used the multi-functional monomer strategy to synthesize MIPs with specific recognition as the adsorbent of PSPE. The method is economical, efficient and environmentally friendly.

MSPE is a new SPE method. It is based on the ability to adsorb and desorb analytes on a magnetic sorbent. Sorption and desorption are performed based on milligrams or micrograms of magnetic sorbent using an external magnetic field, without the need for a series of tedious steps (centrifugation or filtration). Adsorbent particles can be easily separated and collected during the adsorption and desorption process, making the sample pretreatment process more convenient, economical and low-cost. It also avoids the disadvantages of the PSPE cartridge construction [43,44]. Magnetic MIPs as MSPE fillers have been widely used for the detection of many antibiotics [11,45]. Gao et al. [11] synthesized magnetic carbon nanotube dummy MIPs and used them as the filler of MSPE. It is used for the separation and enrichment of SAs. It has been successfully applied to the analysis of SAs in actual fish and shrimp samples.

DSPE involves dispersing the solid sorbent directly into the sample solution, increasing the contact area during the dispersion. After dispersion, the sorbent is centrifuged or filtered from the surface, and once the solid phase has been separated, analytes or interferences adsorbed on the sorbent surface can be easily eluted or eliminated through the addition of sufficient organic solvent. This greatly increases the extraction efficiency and enrichment capacity [46,47]. DSPE with MIPs as adsorbents is widely used in the analysis of antibiotic residues. Lu et al. [23] synthesized MIPs via precipitation polymerization using norfloxacin (NOR) and enrofloxacin (ENR) as double templates. It is used to selectively identify and extract two kinds of antibiotics at the same time. It has been successfully applied to the determination of NOR and ENR in practical water samples.

MSPD is an analytical method for the extraction of analytes from solid, semi-solid and biological matrices. Specifically, the sample is mechanically mixed with an SPE sorbent to produce a semi-dry mixture. It is then used to fill the column, washed with a small amount of reagent to remove impurities and eluted with an appropriate solvent to remove the target compound [48,49]. As a highly selective adsorbent, MIPs have been used in MSPD, which provides strong support for the analysis of antibiotics in complex matrices [49]. Wang et al. [50] synthesized new mixed template MIPs, which can be used as the adsorbent of MSPD to recognize 20 kinds of antibiotics at the same time. This method is fast, simple, specific and sensitive.

SPME is a simple, time-saving and solvent-free technique for sample pretreatment. It is based on the partitioning of analytes between the sample matrix and the polymer film layer. It can obtain the extraction efficiency required for the target components while suppressing other components for enrichment purposes [51,52]. MIP coatings are easy to prepare and have broad application prospects, which are the research focus of SPME coatings [51]. Aguilar et al. [53] synthesized MIPs through precipitation polymerization using TC as a template and used it as the filler of SPME. The results showed that TC, oxytetracycline (OT), chlortetracycline (CT) and doxycycline (DT) had good recognition characteristics and selectivity in the detection and analysis of an actual milk sample.

SBSE is an adsorption technique based on the same principles as those of SPME. It is used to extract and preconcentrate organic compounds in water samples before chromatographic analysis. SBSE has a large preconcentration capacity, requires relatively low sample volumes and can be run overnight without any special requirements. Due to its large volume and surface area, the extraction capacity is higher than that of SPME [54,55]. Because SBSE is not convenient for in vivo or on-site sampling, the application of SBSE based on a MIP coating for antibiotics is less frequent [56]. Cui et al. [56] synthesized MIPs via emulsion polymerization with sulfamonomethoxine (SMM) as a template, as an adsorbent for SBSE. It shows excellent category selectivity for SAs.

PT-SPE is a new form of SPE that uses a pipette tip as a SPE cartridge and requires a small amount of sorbent, thus greatly reducing sample and solvent consumption, saving costs and making the analysis more environmentally friendly. The technique is simple to operate without the need for additional instrumentation. Moreover, it avoids the disadvantages of traditional pretreatment methods. The sorbent in this method is a key factor in the PT-SPE procedure, which determines the extraction efficiency of the analyte [57,58]. MIPs have been used as adsorbents for PT-SPE because of their high stability and specificity [59,60]. Teixeira et al. [60] synthesized MIPs as a PT-SPE adsorbent and combined them with HPLC-UV, which has good sensitivity and accuracy. It was verified that this method may be suitable for the extraction of MALs in complex samples.

## 4. Sensors Based on MIPs

**Figure 3:**
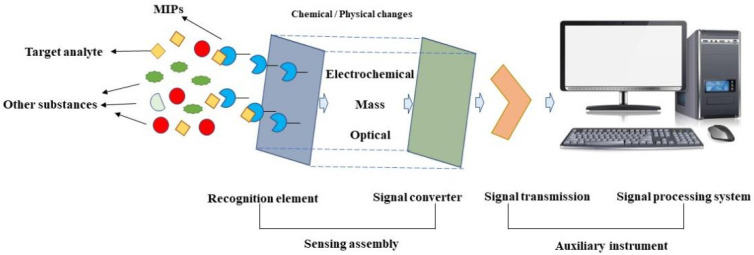
Fundamental construction and principle of MIP-based sensors. Adapted from Ref. [62].

Sensors are important tools in the analytical determination field and mainly consist of a recognition element and a signal converter. The recognition element, also known as the receptor, recognizes and combines the target molecule, while the signal converter converts the target molecule recognized by the recognition element into an output signal. The MIP-based sensors combine MIPs as receptors with sensors and feature molecular recognition specificity, compared with other receptors. Therefore, MIT has become a promising method to improve the target selectivity of chemical/biological sensors [61]. The fundamental construction and principle is schematically illustrated in Figure 3, adapted from Ref. [62]. Moreover, the optical type mainly includes fluorescence, surface-enhanced Raman scattering (SERS) and surface plasmon resonance (SPR), wherein electrochemical sensors and optical sensors are widely used in the analysis of antibiotic residues because of their high sensitivity and fast detection [63,64,65]. 

Electrochemical sensors mainly convert the interaction between the analyte and the electrode surface into analytical signals, and current, voltage and so on will be affected by this effect. The MIP-based electrochemical sensor combines the advantages of MIT and electrochemical analysis and proves that template molecules have excellent repeatability, sensitivity and chemical stability. In the detection and analysis of antibiotics, a MIP-based electrochemical sensor is an important way to analyze antibiotic residues in complex matrices. The electrodes used in electrochemical sensors mainly include glass carbon electrodes and screen-printed carbon electrodes. These have the advantages of high sensitivity, a fast response and low price [64,65,66,67,68,69,70,71]. MIPs are used as recognition elements fixed on the electrode surface in electrochemical sensors because of their high stability and specific recognition [13,71]. Guney et al. [72] synthesized MIPs using the sol-gel method and prepared electrochemical sensors on graphene oxide-modified electrodes (MIP-GO/GCEs). The results showed that the sensor has high sensitivity and selectivity and can be used to detect amoxicillin (AMO) in real samples.

An optical sensor refers to the use of optical devices to convert the specific combination of identification elements and targets into output signals. The main task of MIPs is to quickly bind target biomolecules and perform specific recognition, which is a sensitive recognition element in sensors. The optical unit is used as the signal conversion element to convert the identified biomolecular signal into an optical signal in real time. Because the optical element has a good insulation shielding effect and good anti-interference ability, and has the advantages of simple operation, a fast response speed and high sensitivity. Among them, fluorescent sensors, optical fiber sensors and SPR are most commonly used for antibiotic detection and analysis [14,18,73]. MIPs are combined with the object to be tested as identification elements to achieve the detection purpose [13]. Cheng [74] prepared a novel MIP-based fluorescence sensor using NOR as a template with the reverse microemulsion method for the detection of NOR in actual environmental water samples. The sensor enables the rapid and highly selective detection of NOR.

The mass-sensitive sensor is mainly used to obtain the mass and concentration of the substance to be measured by measuring the small change in the mass of the sensor system or the change in acoustic parameters caused by the change in the mass of the sensor system. It is often used for macromolecular targets with a relatively large mass [75]. Quartz crystal microbalance (QCM) is a kind of quality-sensitive sensor. Based on the principle of quality-sensitive detection, it enables analyte binding to occur on the crystal surface. As a recognition element, MIPs can specifically identify the target analyte to improve selectivity and sensitivity [76]. The combination of the high selectivity of MIPs and high sensitivity of QCM has been used for the detection and analysis of antibiotics [13]. Ayankojo et al. [77] developed a chemical sensor based on MIPs and QCM to detect AMO antibiotics in water samples. Compared with that of other non-templated molecules, the sensor shows good selectivity for the target analyte AMO.

## 5. Applications of MIPs for Antibiotic Analysis

The applications of MISPE and MIP sensors for the analytical determination of antibiotic residues are comprehensively reviewed according to the different kinds of SAs, QNs, BALs, MALs, TCs, AGs and others. Some typical examples of MISPE in antibiotic analysis are listed in Table 1 [10,11,23,40,42,45,49,51,56,59,78,79,80,81,82,83,84,85,86,87,88,89,90,91,92,93,94,95], and MIP-based electrochemical sensors and optical sensors for antibiotic analysis are shown in Table 2 [68,69,70,72,96,97,98,99,100,101,102,103,104,105,106,107] and Table 3 [74,76,108,109,110,111,112,113,114,115,116,117,118,119,120], respectively.

### 5.1. SAs

SAs are a common class of synthetic antimicrobial drugs that are widely used in the farming industry. However, long-term overuse will lead to incomplete degradation, leaving SAs vulnerable to human health risks as residual components in wastewater and entering the environment through farm and municipal wastewater [10,121]. Therefore, it is necessary to establish rapid and effective methods for the extraction and enrichment of trace SAs residues in complex samples [122].

There have been many studies on the application of MISPE in SA detection [10,11,56,78]. Kechagia et al. [10] adopted a multi-template imprinting strategy, with six kinds of SAs, sulfanilamide (SNM), sulfacetamide (SCM), sulfadiazine (SDZ), sulfathiazole (STZ), sulfamerazine (SMZ) and sulfamethizole (SMT), as template molecules. Highly selective MIPs were synthesized via the one-pot sol-gel synthesis approach. The synthesized MIPs were used as PSPE adsorbents to identify six kinds of SAs in milk samples. These were detected and analyzed via HPLC with diode array detection (DAD). The results showed that the limits of detection (LODs) were 1.9–13.3 μg/kg. The approach has the advantages of high selectivity, being green and involving a simple operation. There are many SPE methods based on MIPs for the highly efficient enrichment and separation of various antibiotics in samples that researchers have reported (Table 1).

Wang et al. [78] adopted the dummy template imprinting strategy and took sulfameobenzene (SZ) as the dummy template. MIPs were synthesized through precipitation polymerization and used as an adsorbent of DSPE. Under the optimized conditions, combined with HPLC-UV, SAs in water samples were detected and analyzed. The results showed that the linear range was 1–200 μg/L, the LODs were 0.27–0.64 μg/L, and the recovery was 93.8–102.6%. The method is simple, rapid and suitable for the analysis of trace SAs in complex samples. Cui et al. [56] developed a MISPE method to determine SAs in enriched samples via LC-MS/MS. The recovery is 74–96%, and the detection limit is 1.5–3.4 ng/g. This method has great potential for the rapid separation and enrichment of trace pollutants.

MIP-based sensors have been widely used in SA analysis and detection [68,108,109]. Kurc et al. [108] constructed a highly selective and reusable SPR sensor chip. MIPs synthesized with sulfamethoxazole (SMX) as a template were used as receptors. The surface of the gold SPR chip was coated through drop casting. It was characterized via scanning electron microscopy (SEM), atomic force microscopy (AFM) and Fourier-transform infrared spectrometry (FT-IR). The LOD was 0.0011 µg/L, and the limit of quantification (LOQ) was 0.0034 µg/L. This method can be used to determine SMX in research and industrial applications.

**Figure 4:**
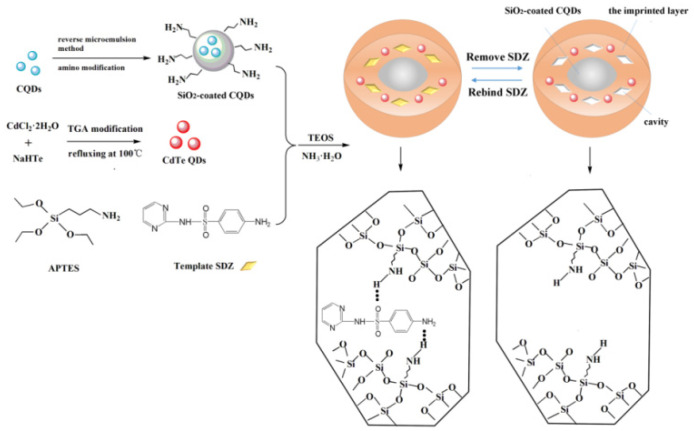
A schematic diagram for the preparation of ratiometric fluorescence nano-sensors [109].

Chen et al. [109] designed a novel double emission surface MIP-based sensor for the specific adsorption and detection of SDZ. A schematic diagram for the preparation of ratiometric fluorescence nano-sensors is shown in Figure 4. The detection of SDZ was successfully achieved in a concentration range of 0.25–20 μmol/L, the LOD was 0.042 μmol/L, and nano-sensors had specific recognition capacity for SDZ over its analogues. In addition, the nano-sensor has been successfully applied to the determination of SDZ in actual water and milk samples with acceptable recovery. This study provides a feasible method for the detection of SDZ. Other researchers also reported the application of MIP-based optical sensors for antibiotic detection and analysis (Table 3). Guo et al. [68] established an electrochemical sensor by using MIPs as identification elements. This was used for the detection and analysis of SAs. The LOD was 1.2 μg/kg. The preparation of the sensor provides a new method for food safety monitoring. 

### 5.2. QNs

QNs comprise a new type of synthetic antibacterial drug, which has the advantages of a broad antibacterial spectrum, good efficacy and minimal side effects, and it is widely used to treat human and animal infectious diseases. However, the long-term use of QNs can cause resistance in animals and result in antibiotic residues in the body [71,123,124]. Therefore, it is very important to monitor the use of QNs.

There are also many studies on the detection and analysis of QNs based on MISPE [23,45,51,59,79]. Zhu et al. [79] adopted the multi-functional monomer imprinting strategy. MIPs were synthesized using ciprofloxacin (CIP) as a template and 1-allyl-3-vinyl imidazole chloride and 2-hydroxyethyl methacrylate as bifunctional monomers. MIPs, as adsorbents of PSPE, are combined with HPLC. The results showed a good linear range within 0.29–1.47 × 10^−5^ µg/L. This has been successfully applied to the separation and enrichment of trace CIP in water, soil and pork samples with recoveries of 87.33–102.50%. The method is green, environmentally friendly and non-polluting, and the functional monomer is an ionic liquid that does not consume organic solvents. Thus, it has great potential for the detection of trace antibiotics in various complex matrices.

**Figure 5:**
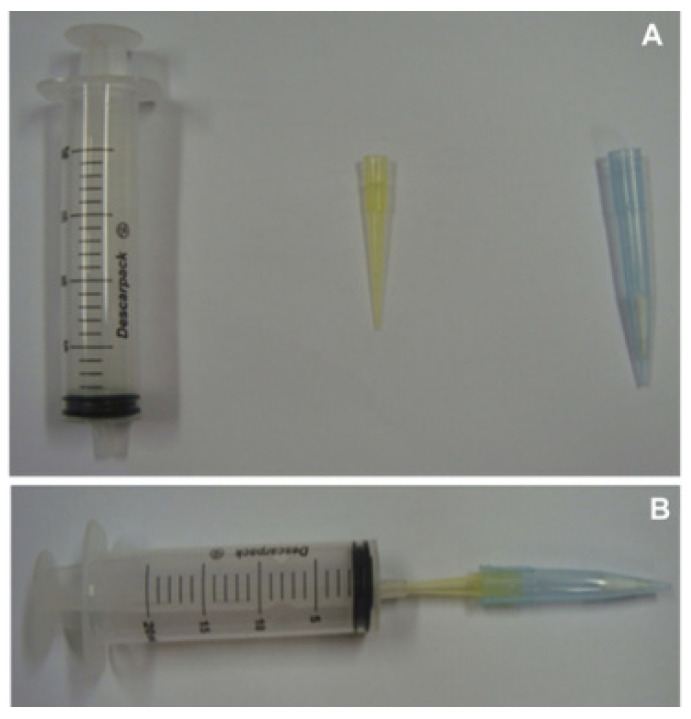
Apparatus employed for PT-MIP-μ-SPE. (**A**) Components of the apparatus and (**B**) apparatus mounted [125].

Hashemi et al. [59] prepared MIPs through an in situ polymerization method with CIP as the template and methacrylic acid (MAA) as a functional monomer; then, they used them as adsorbents for pipette-tip micro solid phase extraction (PT-µSPE) for the detection and analysis of CIP. The schematic diagram of the instrument is shown in Figure 5. The linear range of the method was 5–150 μg/L with an LOD of 1.50 μg/L under optimal conditions. The method has the advantage of low organic solvent usage and a good linear range.

Barahona et al. [51] prepared a new selective MIP for the determination of QNs in environmental water samples using ENR as a template molecule, MAA as a functional monomer, ethylene glycol dimethacrylate (EGDMA) as a cross-linker and 2,2′-azobisisobutyronitrile (AIBN) as an initiator, in combination with SPME. The results were analyzed via HPLC-UV and HPLC-MS/MS under optimal conditions and showed that the recoveries of the method in actual samples of surface water ranged from 9.4 to 24.5% with LODs of 0.1–10 μg/L. The method has the advantage of using less organic solvent in practical applications.

Lu et al. [23] prepared dual template MIPs (dt-MIPs) through precipitation polymerization using NOR and ENR as templates; With MAA as a functional monomer and EDGMA as a cross-linker, the synthesized MIPs were used as the filler of DSPE, the results showed that the LODs of NOR and ENR were 0.22 µg/L and 0.36 µg/L, respectively, and the LOQs were 0.67 µg/L and 0.98 µg/L. Spiked recoveries ranged from 80.9 to 101.0% with satisfactory results. The method has been successfully applied to the detection and analysis of NOR and ENR in lakes, seawater and tap water.

**Figure 6:**
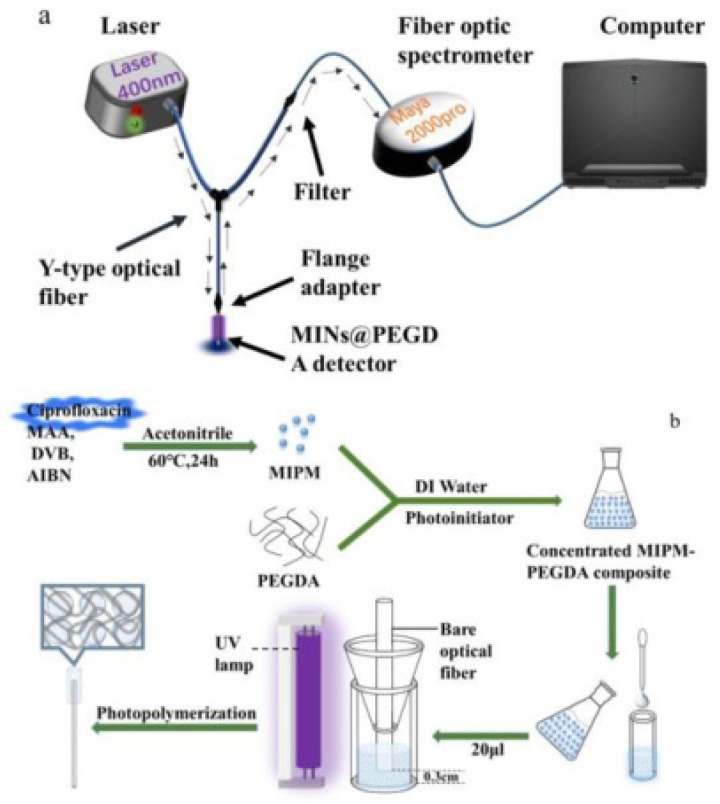
Scheme of MIFOFS and MINs@PEGDA detector ((**a**) Diagram of MIFOFS system; (**b**) outline draft for modification of the MINs@PEGDA detector) [110].

There are also related studies on the detection of QNs using optical/electrochemical sensing [69,74,96,97,98,99,110,111]. Huang et al. [110] developed a novel molecularly imprinted fluorescent optical fiber sensor (MIFOFS) for the rapid detection of CIP. As shown in Figure 6, it was characterized via SEM and transmission electron microscopy (TEM) and analyzed through detection. There was a good linear relationship between CIP and fluorescence intensity with a linear range of 10–500 μmol/L and LOD of 6.86 μmol/L. The sensor has the advantage of being removable and replaceable and can be used for the determination of different antibiotics by using different template molecules and prepared as different detectors for the determination of different antibiotics in environmental water.

Surya et al. [69] established an electrochemical biomimetic sensor for the detection of CIP. A chitosan gold nanoparticle-modified MIP (Ch-AuMIP) was used to modify the glassy carbon electrode (GCE) for preparation of the sensor. The Ch-AuMIP was characterized via SEM, AFM and cyclic voltammetry (CV). The LOD under the optimal conditions was 210 nmol/L, and it had good linearity in the range of 1–100 μmol/L. The sensor has been successfully applied to tap water and milk samples with recoveries in the range of 94–106%, providing a viable method for the detection of CIP in different samples.

Li et al. [98] successfully developed a novel MIP electrochemical sensor for the sensitive detection of lomefloxacin (LFX). The synthesized Fe-doped porous carbon (Fe-PC) was used to modify the gold electrode. By using LFX as a template and *o*-phenylenediamine (*o*-PD) and β-cyclodextrin as a bifunctional monomer, MIP films were prepared on the surface of a Fe-PC modified gold electrode via electro-polymerization. The results showed that there was a good linear relationship between the LFX concentration and current response in the range of 1–120 nmol/L, and the LOD was 0.2 nmol/L. The sensor has been successfully applied to the detection of LFX in real water and milk samples, and the recovery rate was 86.6–105.0%. 

Hammam et al. [99] synthesized MIPs for the detection and analysis of moxifloxacin hydrochloride (MFLX), using MFLX as the template, MAA or 4-vinyl pyridine (4-VP) as the functional monomer, EGDMA as the cross-linker and a mixture of dimethyl sulfoxide and acetonitrile as the porogen. The average recovery of the sensor with human urine samples is 96.6–102.8%.

### 5.3. BALs

BALs are one of the most used antibiotic species today and are associated with highly resistant strains of *Staphylococcus, Escherichia coli* and *Klebsiella*, which are capable of producing extend-spectrum β-lactamases. These have become a serious problem in antimicrobial chemotherapy and pose a significant threat to human health [126].

Pourtaghi et al. [80] prepared MIPs using penicillin G (PEN-G) as a template, MAA as a functional monomer and EGDMA as a cross-linker, as an adsorbent for SPE, combined with HPLC-UV, to detect PEN-G in milk samples. The results showed that the LOD was 2 ng/mL and the relative recoveries were 81–90%. It has been successfully applied to commercial sterilized milk samples for the detection of PEN-G residues. Others also reported the determination of BALs using MISPE combined with LC-MS/MS [80,81,82,83].

Tian et al. [81] used a surface imprinting technique to prepare MIP microspheres with the ability to specifically recognize water-soluble molecules using ampicillin (AMP) as a template, acrylamide as a functional monomer and EGDMA as a crosslinker. It was used as an SPE filler and combined with HPLC. AMP in egg was successfully separated and enriched. The recoveries were 91.5–94.9%. It can be reused to 10 times. This method is more sensitive and rapid for the determination of AMP in food.

MIP-based sensors are also used for the detection of BALs [70,72,100,112,113]. Bereli et al. [112] prepared SPR and QCM sensors using MIT. MIPs were synthesized on the surface of SPR and QCM chips via ultraviolet (UV) polymerization, as receptors to recognize traces of AMO, and were analyzed through HPLC-MS/MS, with a linear range of 0.1–10 ng/mL. The sensor was successfully applied to eggs.

Bakhshpour et al. [113] prepared MIP-based SPR sensors using nano-silver. This sensing system was also successfully used for PEN-G recognition in milk samples. The linear concentration range was 0.01–10 ng/mL under optimal conditions, and the sensor is simple and sensitive without any complex coupling process.

### 5.4. MALs

MALs are a class of antibiotics containing 12–16 carbon atoms in their structure and are used to treat different types of infectious diseases and to promote growth in animals, while the excessive use of MALs can lead to environmental pollution and thus harm human health [127].

The detection and analysis of MALs based on MISPE has also been studied by others [42,84,85]. Song et al. [84] prepared MIPs via precipitation polymerization using tulathromycin (TUL) as a template and MAA as a functional monomer and used them as adsorbents for DSPE. Combined with HPLC-MS, seven MALs in pork were determined simultaneously with average recoveries between 68.6 and 95.5% and LODs of 0.2–0.5 μg/kg, indicating that the method was rapid, effective and selective.

Song et al. [85] synthesized MIPs using tylosin (TYL) as a template and MAA as a functional monomer via a bulk polymerization method as absorbents for MISPE. The method provides a feasible approach for the determination of MALs in water samples. The results showed that its LODs were 1.0–15.0 ng/L, its LOQs were 3.0–40.0 ng/L, and the recovery was 62.6–100.9%.

It has been reported that MIP-based sensors can be used for the detection and analysis of MALs [101,102,103]. Hu et al. [101] prepared an electrochemiluminescence sensor using MIPs with azithromycin (AZM) as the template and using it as the recognition element. The factors affecting AZM were systematically optimized, with good linearity between 1.0 × 10^−10^ and 4.0 × 10^−7^ mol/L and an LOD of 2.3 × 10^−11^ mol/L with urine under optimal conditions, as well as with a spiked recovery of 98.4–113.5%; the method allows for the rapid and highly sensitive determination of trace AZM in complex samples.

**Figure 7:**
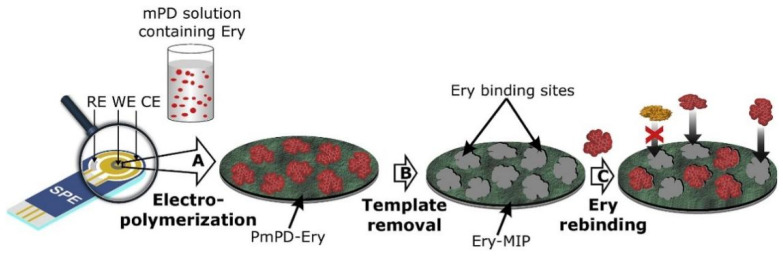
Schematic of the protocols for Ery-MIP film formation on the gold working electrode of a screen-printed electrode [102].

Ayankojo et al. [102] constructed a portable electrochemical sensor using erythromycin (Ery) as a template and MIPs as the recognition element. As shown in Figure 7. Ery-MIP was generated directly on screen-printed electrodes via the electrochemical polymerization of m-PD, and after optimizing the performance of the sensor, it was characterized through CV and electrochemical impedance spectroscopy (EIS). The analysis via HPLC-MS showed that the recoveries of the LOD and LOQ in real tap water samples were 0.1 nmol/L and 0.4 nmol/L, respectively.

### 5.5. TCs

TCs are widely used for the prevention and treatment of animal diseases due to their spectral antimicrobial activity and low cost [49]. However, excessive use can lead to trace levels of TC residues in foods of animal origin. This may cause allergic reactions in susceptible populations and consequently resistance to the drugs [128].

**Figure 8:**
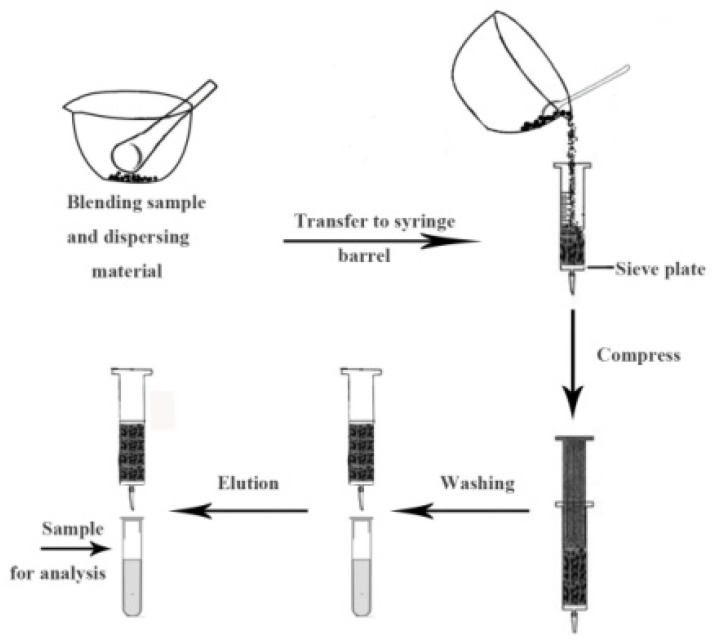
The extraction procedure of MSPD [49].

Research to detect TCs based on MISPE is very extensive [49,86,87,88,89]. Wang et al. [49] synthesized novel MIPs that selectively recognize TCs in milk powder using a metal-organic framework (MOF) as support materials, TC as a template molecule and 3-aminophenylboronic acid as a functional monomer and cross-linker and used them as adsorbents for MSPD. As shown in Figure 8. TCs extracted from milk powder were determined via UHPLC-MS. The results showed that the LODs were 0.217–0.318 ng/g, the recoveries were between 84.7 and 93.9%, and the method has been successfully applied to real samples.

Ma et al. [86] polymerized dimethylamine TC-MIPs on the surface of MOF. It was used as an adsorbent for DSPE for the detection of TCs in chicken meat. When combined with HPLC, it showed high absorption capacities and high recoveries of TCs. The LODs for the seven drugs were in the range of 0.2–0.6 ng/g and the LOQs were in the range of 0.5–2.0 ng/g. This method enables the rapid and accurate determination of TCs in meat samples. In addition, Zeng [87], Guo et al. [88], Huang et al. [89] and others also studied the use of MISPE to detect TCs.

There are a number of related reports on MIP-based electrochemical/fluorescence sensing for the detection and analysis of TCs [104,105,114,115,116]. Wei et al. [114] established a carbon quantum dot (CQD) molecularly imprinted fluorescence sensor with the CQDs surface modified by acrylic acid, which was characterized via TEM and FT-IR. The results showed good linearity in the range of 1.0–60 μmol/L with an LOD of 0.17 μmol/L under optimal conditions. The sensor has good selectivity and high sensitivity for TCs, providing a feasible method for the detection of trace TCs.

**Figure 9:**
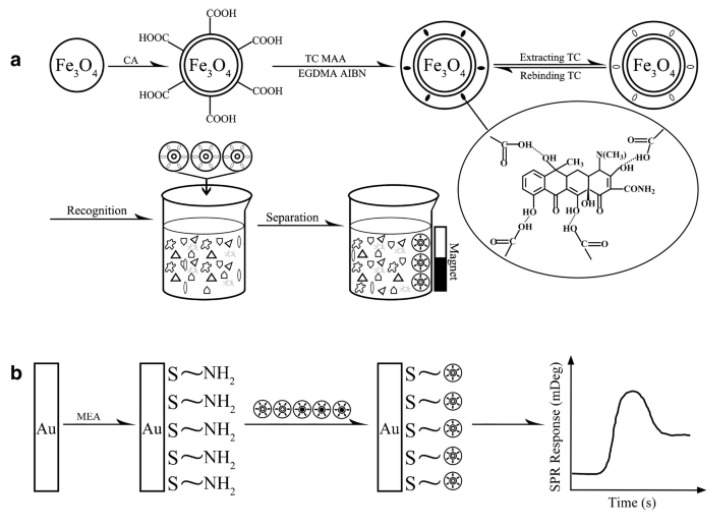
Schematic diagram of the synthesis of MMIP NPs for the recognition and separation of tetracycline (TC) (**a**) and MMIP NP-enhanced SPR for TC detection (**b**) [115].

Gao et al. [115] established an in situ detection method for TCs. It is based on the combination of the extraction of TCs using magnetic MIPs (MMIPs) and detection via SPR, as schematically shown in Figure 9. The MMIPs showed good linearity in the range of 5.0–100 pg/mL with an LOD of 1.0 pg/mL. The sensor showed good selectivity for TCs and has been successfully applied to milk samples for the detection and analysis of TCs with recoveries of 95.7–104.6%.

Zeb et al. [105] prepared an electrochemical sensor composed of magnetic nanoparticles and MIPs for the detection of TCs. After optimizing the performance of the sensor, CV and differential pulse voltammogram (DPV) detection analysis showed that there was a good linear relationship in the range of 5.0 × 10^−7^ to 2.0 × 10^−5^ mol/L, and the LOD was 1.5 × 10^−7^ mol/L. The sensor has been successfully applied to the detection and analysis of TCs in milk samples with a recovery rate of 93–103%.

### 5.6. AGs

AGs have been widely used in animal husbandry to inhibit the growth and reproduction of bacteria and promote the growth of animals because of their good broad-spectrum antibacterial activity [129]. However, the excessive use of AGs can pose a threat to the environment and human health [90], and thus, there is an urgent need to establish an efficient and rapid method to detect AGs.

**Figure 10:**
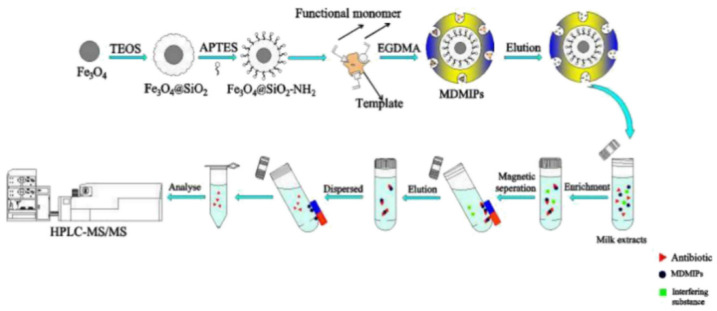
The schematic representation of the proposed MSPE process based on MDMIPs for AGs [90].

Cao et al. [90] used a dummy template imprinting strategy to synthesize magnetic polymers via surface imprinting using raffinose (RAF) as the template molecule, MAA as the functional monomer and trimethylolpropane triacrylate as the crosslinker, with the MIPs polymerizing on the surface of Fe_3_O_4_ for easy removal of the template. A schematic representation of the MSPE process based on magnetic dummy template-MIPs (MDMIPs) for AGs is shown in Figure 10. The MIPs were also used as adsorbents for MSPE and were successfully applied to the analysis of atomic absorption spectroscopy in milk in combination with HPLC-MS/MS, with an LOD of 3.6–9.6 μg/kg and recoveries of 82.6–114.1%. The method was highly sensitive and reproducible, providing a good idea for the detection and analysis of AGs in complex samples.

Zhang et al. [40] used a dummy template imprinting strategy, using RAF as the dummy template. Novel dummy MIPs (DMIPs) of AGs were synthesized via precipitation polymerization, and an effective method for the determination of AGs in aqueous samples based on PSPE coupled with HPLC-MS under optimal conditions was established, with LODs of 0.006–0.6 ng/mL and recoveries of 70.8–108.3%. The results indicate that DMIPs have good potential for the detection and analysis of AGs in environmental water samples. Others have also studied the detection and analysis of MISPE for AGs [91]. See Table 1 for specific examples.

**Figure 11:**
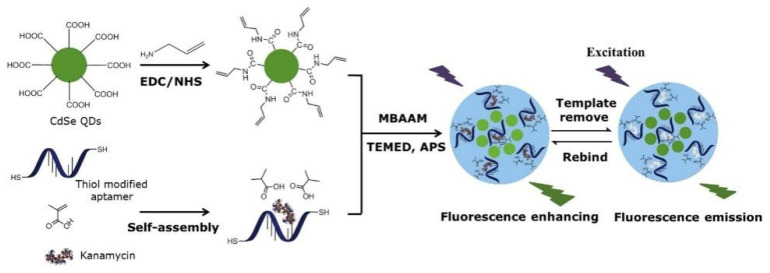
Schematic illustration of the preparation and recognition process for the fluorescent aptamer-functionalized MIPs [117].

As one of the AGs, kanamycin (KAN) is widely used to treat infectious diseases caused by Gram-negative and positive bacteria [117]. Geng et al. [117] prepared an alternative strategy for functionalizing MIP with fluorescent aptamers, used for the highly specific detection of KAN. This technology uses CdSe quantum dots (QDs) as supports, thiol-modified aptamers and MAA as functional monomers and KAN as templates to provide surface imprinting in an aqueous solution. The schematic diagram is shown in Figure 11. MIP can play a role by using the dual recognition of the adapter and the imprinting cavity of the KAN fluorescence sensor. The aptamer was in a polymer matrix using the “thiol-ene”, which is green, environmentally friendly and efficient. The results were investigated via TEM and FT-IR and showed good linearity in the range of 0.05–10.0 μg/mL with an LOD of 0.013 μg/mL. The sensor was successfully applied to the detection of KAN in milk and water samples with satisfactory results.

Zhang et al. [118] prepared MIP-based SPR sensors by using a surface immobilized initiator approach with KAN as the template, 4-vinylbenzeneboronic acid as a functional monomer and EGDMA as a cross-linking agent. It was characterized via SEM and FT-IR under optimal conditions, and the results showed good linearity in the range of 1.00 × 10^−7^–1.00 × 10^−5^ mol/L. It was also successfully applied to the detection of KAN in honey and milk powder. The LODs were 1.20 × 10^−8^ mol/L and 4.33 × 10^−8^ mol/L, respectively. The results demonstrated that the sensor has the advantages of stability and high sensitivity for the detection of KAN in a complex matrix.

Bi et al. [106] used nanoimprinting to establish an electrochemical aptamer sensor for the detection of KAN. After CV detection and analysis, the concentration of KAN showed a good linear relationship with an electrochemical signal strength in the range of 10–500 nmol/L, and the LOD was 1.87 nmol/L. The sensor can be used to detect KAN in aqueous solutions and milk, and its selectivity, stability and reproducibility are acceptable.

### 5.7. Others

Antibiotics are continuously released and persistent in the environment, resulting in the detection of antibiotics in almost every environmental matrix [130]. Their side effects have an impact on human health. For example, chloramphenicol (CAP) is a broad-spectrum antibiotic with good antibacterial performance, but it has been forbidden to be used in animal-derived food due to its considerable toxicity and side effects [131]. Thus, it is vital to establish analytical methods for residue detection to detect antibiotics.

The detection and analysis of other kinds of antibiotics based on MISPE has also been studied by others [92,93,94,95]. For example, Lian et al. [92] prepared MIPs using CAP as the template molecule, MAA as the functional monomer and EGDMA as a cross-linking agent via precipitation polymerization. They evaluated their morphology, capacity and selectivity and used them as adsorbents for PSPE in combination with HPLC-DAD to enrich and purify CAP from complex seawater samples, with results showing recoveries of 81–90%. This method has good potential for the detection and analysis of CAP in complex matrices.

Li et al. [93] prepared MMIPs with CAP as the template molecule, MAA as the functional monomer and EGDMA as the cross-linking agent via suspension polymerization on Fe_3_O_4_ magnetic nano-surfaces. The adsorption of MMIPs was investigated by combining experiments with an external magnetic field as an adsorbent for SPE for the simple and rapid separation of MMIPs, and combined with HPLC-UV assay analysis, it showed an LOD of 10 μg/L and recoveries of 95.31–106.89%. This method is effective and simple for the detection of CAP in milk and eggs.

There are related reports on the detection and analysis of CAPs based on sensors based on MIPs [76,107,119,120]. For example, Amiripour et al. [119] established an optical sensor for the detection of CAP by overlaying CAP-MIPs onto a luminescent zirconium MOF (MIP/Zr-MOF). The framework was studied via TEM and field emission SEM. It was shown that Zr MOF in a porous structure exhibits the intentional recognition of CAP. The LOD was 0.013 μg/L, and it showed good recovery in the determination of real samples of milk and honey. The results showed that the sensor is a practical method for the determination of CAP residues.

**Figure 12:**
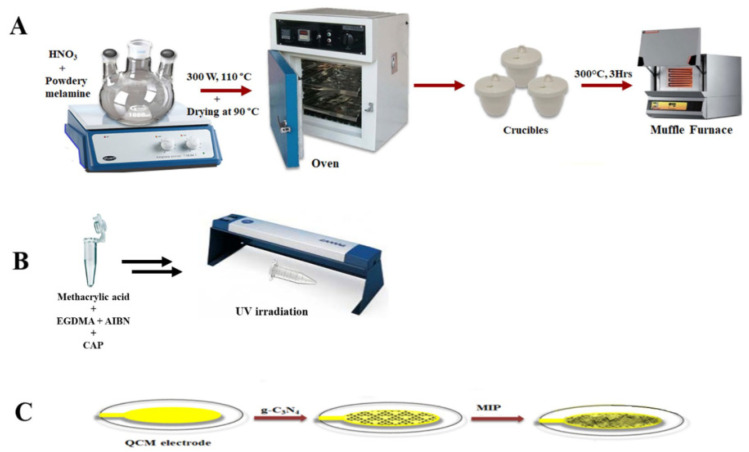
Schematic diagram illustrating the (**A**) synthesis of g-C_3_N_4_ nanosheets, (**B**) synthesis of MIP (**C**) and design of the mass-sensitive nanointerfaces for the detection of chloramphenicol [76].

Shaheen et al. [76] synthesized two-dimensional graphitic carbon nitride nanosheets (g-C_3_N_4_) using a microwave-assisted method and mixed them with MIPs prepared using CAP as a template to establish a novel mass-sensitive sensor, as shown in Figure 12. The nano-interface was generated by modifying a QCM and characterized via AFM and SEM, showing an LOD of 177 μmol/L. The sensor has good sensitivity and selectivity, providing a new method for the practical determination of CAP residues in complex samples.

## 6. Conclusions and Perspectives

To conclude, we review the emerging preparation techniques for MIPs for the detection of antibiotics, the principle and classification of MISPE, the principle and classification of MIP-based sensors, and the typical applications of MIPs for SAs, QNs, BALs and other antibiotics. By summarizing these studies, perspectives can be proposed as follows. (1) MIPs prepared using emerging technologies greatly improve the performance of MISPE and MIP-based sensors. (2) MIPs provide a feasible material for eliminating matrix interference and efficiently enriching trace antibiotics. (3) The new MIPs for detecting antibiotics are used as the filler of SPEs and the recognition element of the sensor, which effectively solves the problems of the low binding ability of traditional MIPs and template leakage. (4) The development of MISPE makes it possible to detect trace antibiotics more effectively. (5) The construction strategy for MIP-based sensors should be greatly developed, and this can be used to solve the problems of multiple pollutants at the same time and the problem of unsustainable imprinting [132,133]. (6) The green MIPs follow green principles, are closely related to sustainability and have good application prospects for sample pretreatment [134,135,136,137]. (7) Great explorations are imperative to find new enrichment materials and develop new methods for green MIP preparation. In addition, it is necessary to actively promote the large-scale production of MISPE and MIP-based sensors to push forward their greater development and wider applications.

## Figures and Tables

**Table 1:** Applications of MISPE for antibiotic analysis.

Types of Antibiotics	SPE Mode	Analyte/Number	Imprinting Technique	Template	Polymerization Method	LOD	Detection Technique	Real Sample	Recovery/%	Ref.
SAs	PSPE	SAs/6	multi-template imprinting strategies	SNM, SCM, SDZ, STZ, SMZ, SMT	sol-gel polymerization	1.9–13.3 μg/kg	HPLC-DAD	milk	-	[10]
	MSPD	SAs/8 QNs/8 TCs/4	-	SB, PA, TC	-	0.5–3.0 ng/g	UPLC	pork	74.5–102.7	[50]
	DSPE	SAs/4	dummy template imprinting	SZ	precipitation polymerization	0.27–0.64 μg/L	HPLC-UV	water	93.8–102.6	[78]
	SBSE	SAs/4	-	SMM	emulsion polymerization	1.5–3.4 ng/g	LC–MS/MS	feed	80.6–89.7	[56]
	MSPE	SAs/14	dummy template imprinting	SB	-	0.1 μg/kg	UPLC-MS/MS	fish, shrimp	90.2–99.9	[11]
QNs	PSPE	QNs/3	multi-functional monomer imprinting strategies	CIP	-	0.11 μg/L	HPLC	water, soil and pork	87.33–102.50	[79]
	PT-μSPE	CIP/1	-	CIP	in situ polymerization	1.50 μg/L	Spectrophotometry	sea water	86.9–99.6	[59]
	SPME	FQs/4	-	ENR	-	0.1–10 μg/L	HPLC-UV, HPLC-MS/MS	water	9.4–24.5	[51]
	DSPE	FQs/2	dual-template imprinting strategies	NOR, ENR	precipitation polymerization	0.22 μg/L, 0.36 μg/L	HPLC	lake, ocean, tap water	80.9–101.1	[23]
	MSPE	FQs, TC, SA/4	-	CIP	ATRP	-	HPLC	-	-	[45]
BALs	PSPE	penicillin G/1	-	PEN-G	-	2 ng/mL	HPLC-UV	milk	81–90	[80]
	PSPE	BALs/4	surface imprinting	AMP	-	-	HPLC	food	91.5–94.9	[81]
	PSPE	BALs/3	-	PEN-G	photopolymerization	-	LC-MS	-	-	[82]
	PSPE	BALs/5	multi-template imprinting strategies	AMO, CFX, CFZ, PEN-G, OXA	-	0.24–0.56 μg/L	HPLC	lake water, pond water	91.3–110.1	[83]
MALs	DSPE	MALs/7	-	TUL	precipitation polymerization	0.2–0.5 μg/kg	HPLC-MS/MS	pork	68.6–95.5	[84]
	PSPE	MALs/10	-	TYL	bulk polymerization	1.0–15.0 ng/L	LC-MS/MS	water	62.6–100.9	[85]
	PSPE	KITA/1	multi-functional monomer imprinting strategies	KITA	-	0.1 mg/L	HPLC	soil, water	92.3–108.8	[42]
TCs	MSPD	TCs/3	-	TC	-	0.217–0.318 ng/g	UHPLC-MS/MS	milk powder.	84.7–93.9	[49]
	SPME	TCs/4	-	TC	precipitation polymerization	-	LVSS-CE	milk	-	[53]
	DSPE	TCs/7	surface imprinting	MC	-	0.2–0.6 ng/g	UHPLC-PDA	chicken muscle	69.6–94.7	[86]
	PSPE	TCs/4	dummy template imprinting	TC, OTC, CTC, DC	-	0.74, 0.67 0.92, 0.95 μg/L	HPLC	river water, pond water	82.7–103.3	[87]
	MSPE	TCs/2	surface imprinting	TCs	-	0.39 mg/L	HPLC	-	-	[88]
	PSPE	TCs/3	-	TC, CTC, DC	in situ polymerization	3.0–5.0 μg/kg	HPLC-FLD	egg	86.4–94.2	[89]
AGs	MSPE	AGs/6	dummy template imprinting, surface imprinting	RAF	-	3.6–9.6 μg/kg	HPLC-MS/MS	milk	82.6–114.1	[90]
	DSPE	AGs/6	dummy template imprinting	RAF	precipitation polymerization	0.006–0.6 ng/mL	HPLC-MS/MS	water	70.8–108.3	[40]
	PSPE	AGs/11	-	AGs	-	1.0–10.0 μg/kg	UPLC-MS/MS	aquatic products	78.4–109.6	[91]
others	PSPE	CAP/1	-	CAP	precipitation polymerization	5 ng/L	HPLC-DAD	sea water	81–90	[92]
	PSPE	CAP/1	multi-functional monomer imprinting strategies	CAP	suspension polymerization	10 μg/L	HPLC-UV	food	95.31–106.89	[93]
	PSPE	LIN/1	-	LIN		0.02 μg/mL	HPLC-UV	milk	80–89	[94]
	MSPE	LIN, AMO, OXYT, CLIN/4	surface imprinting	LIN	ATRP	15.0 ng/g	HPLC-UV	milk	94.3–98.2	[95]

Note: “-” indicates “none”; SB: sulfabenzamide; PA: pipemidic acid; LVSS-CE: large volume sample stacking capillary electrophoresis; KITA: kitasamycin; PDA: photo-diode array; FLD: fluorescence detection; DAD: diode array detection; CLIN: clindamycin; OXYT: oxytetracycline hydrochloride; CFZ: cefazolin; CFX: cefalexin; OXA: oxacillin.

**Table 2:** Applications of MIP-based electrochemical sensors in antibiotic detection.

Types of Antibiotics	Templates/Analytes	Function Monomers	Electrode	Detection Mode	Linear Range	LOD	Real Sample	Recovery/%	Ref.
SAs	SMX	MAA	GCE	CV	5 μg/kg–1 mg/g	1.2 μg/kg	food	-	[68]
QNs	CIP	MAA	GCE	CV	1–100 μmol/L	210 nmol/L	water	94–106	[69]
	PEF	-	GCE	DPV	5.0 × 10^−7^–2.0 × 10^−5^ mol/L	1.6 × 10^−8^ mol/L	milk	-	[96]
	NOR	MAA	Ag/AgCl	CV, DPV	0.003–3.125 μmol/L	1.58 nmol/L	-	97.36–109.58	[97]
	LFX	*o*-PD	Fe-PC/Au	CV, DPV	1–120 nmol/L	0.2 nmol/L	water, milk	86.6–105.0	[98]
	MFLX	MAA/4-VP	Ag/AgCl	-	1.0 × 10^−5^–1.0 × 10^−2^ mol/L	1.7 × 10^−6^ mol/L	urine	96.6–102.8	[99]
BALs	AMO	MAA	Ag/AgCl	DPV, EIS	5–1500 × 10^−11^ mol/L	9.2 × 10^−12^ mol/L, 8.3 × 10^−12^ mol/L	-	-	[100]
	AMO	APTES, PTES	GO/GCE	CV, DPV	5.0 × 10^−10^–9.1 × 10^−7^ mol/L	2.94 × 10^−10^ mol/L	-	-	[72]
	AMP	NNDMA	Fe_3_N-Co_2_N/CC	DPV	5.56 × 10^−9^–9 × 10^−3^ mol/L	3.65 × 10^−10^ mol/L	milk	97.06–102.43	[70]
MALs	Ery/AZM	MAA	Ag/AgCl	CV, DPV	1.0 × 10^−10^–4.0 × 10^−7^ mol/L	2.3 × 10^−11^ mol/L	-	98.4–113.5	[101]
	Ery	*m*-PD	Ag/AgCl	CV, EIS	-	0.1 nmol/L	tap water	91–102	[102]
	AZY	4-ABA	SPCE	CV, EIS	0.5–10.0 μmol/L	0.08 μmol/L	water	-	[103]
TCs	TC	4-ATP	GCE	CV, EIS	2.0 × 10^−8^–3.0 × 10^−8^ mol/L	1.5 × 10^−9^ mol/L	-	97. 9–106	[104]
	TC	AA	Ag/AgCl	CV, DPV	5.0 × 10^−7^–4.0 × 10^−5^ mol/L	1.5 × 10^−7^ mol/L	milk	93–103	[105]
AGs	KAN	-	Ag/AgCl	CV	10–500 nmol/L	1.87 nmol/L	milk	-	[106]
others	CAP	EBT	carbon screen-printed electrodes	CV, EIS	-	-	-	-	[107]

Note: “-” indicates “none”; PEF: pefloxacin; NNDMA: N-N-dimethyl bisacrylamide; APTES: (3-Aminopropyl) triethoxysilane; PTES: phenyltriethoxysilane; AZY: azithromycin; 4-ABA: 4-aminobenzoic acid; 4-ATP: 4-aminothiophenol; SPCE: screen-printed carbon electrode; Fe-PC: Fe-doped porous carbon; CC: carbon cloth; *m*-PD: *m*-phenylenediamine; EBT, Eriochrome black T.

**Table 3:** Application of MIPs based optical and mass sensors in antibiotic detection.

Types of Antibiotics	Detection Technology	Imprinting Technique	Templates	Polymerization Methods	Linear Range	LOD	Recovery (%)	Real Sample	Ref.
SAs	SPR	-	SMX	-	-	0.0011 μg/L	97.2–99.5	milk	[108]
	fluorescence nano-sensors	surface imprinting	SDZ	-	0.25–20 μmol/L	0.042 μmol/L	79.3–101.2	water, milk	[109]
QNs	fluorescent optical fiber sensor	-	CIP	-	10–500 μmol/L	6.86 μmol/L	-	water	[110]
	fluorescence sensor	-	NOR	-	1.0–100.0 μg/L	0.35 μg/L	93.8–99.3	chicken meat, milk	[111]
	fluorescence sensor	-	NOR	reverse microemulsion	3.82–150 nmol/L	3.82 nmol/L	-	-	[74]
BALs	SPR	-	AMO	UV polymerization	0.1–10 ng/mL	0.0005 ng/mL	-	egg	[112]
	SPR	-	PEN-G	-	0.01–10 ng/mL	-	-	milk	[113]
TCs	fluorescence sensor	-	TC	precipitation polymerization	1.0–60 μmoL/L	0.17 μmoL/L	96–105.6	water	[114]
	SPR	surface imprinting	TC		5.0–100 pg/mL	1.0 pg/mL	95.7–104.6	milk	[115]
	fluorescence sensor	-	OTC	precipitation polymerization	0–80 μmol/L	3.5 nmol/L	99.93–100.20	milk	[116]
AGs	fluorescence sensor	surface imprinting	KAN	-	0.05–10.0 μg/mL	0.013 μg/mL	-	water	[117]
	SPR	surface imprinting	KAN	-	1.00 × 10^−7^–1.00 × 10^−5^ mol/L	1.20 × 10^−8^ mol/L, 4.33 × 10^−8^ mol/L	-	honey, milk powder	[118]
Others	fluorescence sensor	surface imprinting	CAP	-	0.16–161.56 μg/L	0.013 μg/L	96–105	milk, honey	[119]
	mass sensor	-	CAP	free radical polymerization	-	177 μmol/L	90–98	-	[76]
	fluorescence sensor	-	CAP	reversed-phase microemulsion	1.50 × 10^−3^–1.50 × 10^−2^ μmol/L	12.83 nmol/L	90.02–102.53	crucian carp	[120]

Note: “-” indicates “none”; OTC: oxytetracycline.
